# Modeling and prediction of copper removal from aqueous solutions by nZVI/rGO magnetic nanocomposites using ANN-GA and ANN-PSO

**DOI:** 10.1038/s41598-017-18223-y

**Published:** 2017-12-21

**Authors:** Mingyi Fan, Jiwei Hu, Rensheng Cao, Kangning Xiong, Xionghui Wei

**Affiliations:** 1 Guizhou Provincial Key Laboratory for Information Systems of Mountainous Areas and Protection of Ecological Environment, Guizhou Normal University, Guiyang, 550001 Guizhou China; 2 Cultivation Base of Guizhou National Key Laboratory of Mountainous Karst Eco-environment, Guizhou Normal University, Guiyang, 550001 Guizhou China; 3 Department of Applied Chemistry, College of Chemistry and Molecular Engineering, Peking University, Beijing, 100871 China

## Abstract

Reduced graphene oxide-supported nanoscale zero-valent iron (nZVI/rGO) magnetic nanocomposites were prepared and then applied in the Cu(II) removal from aqueous solutions. Scanning electron microscopy, transmission electron microscopy, X-ray photoelectron spectroscopy and superconduction quantum interference device magnetometer were performed to characterize the nZVI/rGO nanocomposites. In order to reduce the number of experiments and the economic cost, response surface methodology (RSM) combined with artificial intelligence (AI) techniques, such as artificial neural network (ANN), genetic algorithm (GA) and particle swarm optimization (PSO), has been utilized as a major tool that can model and optimize the removal processes, because a tremendous advance has recently been made on AI that may result in extensive applications. Based on RSM, ANN-GA and ANN-PSO were employed to model the Cu(II) removal process and optimize the operating parameters, e.g., operating temperature, initial pH, initial concentration and contact time. The ANN-PSO model was proven to be an effective tool for modeling and optimizing the Cu(II) removal with a low absolute error and a high removal efficiency. Furthermore, the isotherm, kinetic, thermodynamic studies and the XPS analysis were performed to explore the mechanisms of Cu(II) removal process.

## Introduction

A wide range of hazardous heavy metals (such as copper, arsenic, chromium, lead, cadmium, mercury and zinc) have been discharged into water bodies due to the rapid growth of various industries^1–3^. The heavy metals pose a significant threat to human health and ecological systems owing to their non-biodegradability, high toxicity and easy accumulatio^4,5^. Among them, copper (Cu) is a toxic heavy metal that has been applied in a variety of industries, such as electroplating, architecture, printed circuit boards and machined products^6^. Although Cu is an important trace element needed by humans for enzyme synthesis, tissue and bone development, it is reported that intake of excessive Cu(II) by human leads to severe mucosal irritation and corrosion, widespread capillary damage, hepatic and renal damage^7^. Therefore, the remediation of Cu(II) pollution in the water environment has become a significant issue to human beings.

Various technologies in recent years have been utilized to remove heavy metals from wastewater, which include adsorption, electrolysis, reverse osmosis, membrane separation and chemical precipitation. Among these technologies, adsorption has been extensively employed because of its simple operation, high efficiency and low cost^8–12^. Graphene is a basic unit for construction of carbonaceous materials, which is composed of two-dimensional sp^2^ carbon network with a honeycomb crystal structure^13^. It also has a large specific surface area (about 2620 m^2^/g), which renders the sheet a good choice for supporting nanoparticles. Graphene oxide (GO) is a lamellar flexible material containing a variety of functional groups (C=C, C=O, OH and C-O-C) on its basal plane and on the edges of its sheet^14^. These oxygen-containing groups do not only improve the adsorption ability for heavy metals, but also enhance the dispersivity of GO^15–17^. Graphene-based magnetic nanocomposites, such as reduced graphene oxide-supported nanoscale zero-valent iron (nZVI/rGO), have recently been shown to be effective in wastewater treatment with a higher efficiency than either of their pure components^18–20^.

Modeling is a proven and accepted engineering approach that can help understand the removal processes^21^. However, modeling removal processes by conventional mathematical models (mechanistic models) are costly and time-consuming due to a broad range of experiments required. Moreover, the processes of wastewater treatment are highly complex, which are affected by various operating parameters and removal mechanisms. Thus, it is difficult to model and optimize the removal processes by using conventional mathematical models^22^. Modeling wastewater treatment processes is recently encouraged by using empirical models, including least squares support vector machines, response surface methodology (RSM) and artificial neural networks (ANNs). RSM is a commonly adopted statistical method for building quadratic models and optimizing the process parameters, and its important advantage is that it requires less number of experiments to be performed. ANNs inspired by biological neurons belong to artificial intelligence (AI) techniques, which have recently experienced a tremendous advance in various applications, e.g., intelligent search, autonomous driving, big data, pattern recognition and robotics^23^. ANNs (the black box models) can model ill-defined and non-linear problems since they can be developed solely from the input and output data without any detailed knowledge of the removal processes^24^. ANNs combined with RSM can be considered as an effective approach when the removal processes are complex and require a large number of experiments and a high consumption of chemical reagents^25^.

Meta-heuristic optimization algorithms, such as genetic algorithm (GA), particle swarm optimization (PSO) and ant colony algorithm, were originated from social behavior or natural phenomena, which have been utilized to carry out the optimization of removal processes^26,27^. GA is used for finding precise or approximate optimal operating parameters of removal processes based on the fundamental of evolution from natural selection^28^. Another well-known optimization algorithm is PSO, which was inspired by the behavior of a bird flock^29^. Both GA and PSO are now frequently applied in the optimization of removal processes because they do not easily get trapped in a local minimum^23,27,30–32^.

To our knowledge, limit studies have hitherto been reported concerning the modeling and optimization for the Cu(II) removal process using RSM, ANN-GA and ANN-PSO. In this work, the nZVI/rGO magnetic nanocomposites were prepared by chemical deposition method and characterized by scanning electron microscopy (SEM), transmission electron microscopy (TEM), superconduction quantum interference device (SQUID) magnetometer and X-ray photoelectron spectroscopy (XPS). Then, the Cu(II) removal process was modeled and optimized by using RSM, ANN-GA and ANN-PSO in order to obtain the maximum removal efficiency. The performance of these three models was evaluated based on the correlation coefficient (R^2^) and the absolute error. Analysis of variance (ANOVA) and sensitivity analysis were carried out to investigate the relative importance of independent variables. The isotherm, thermodynamics and kinetics studies were also performed to investigate the behavior of removal process. Finally, the mechanisms for the Cu(II) removal process were explored based on the XPS results.

## Results and Discussion

### Characterization of the nZVI/rGO magnetic nanocomposites

**Figure 1:**
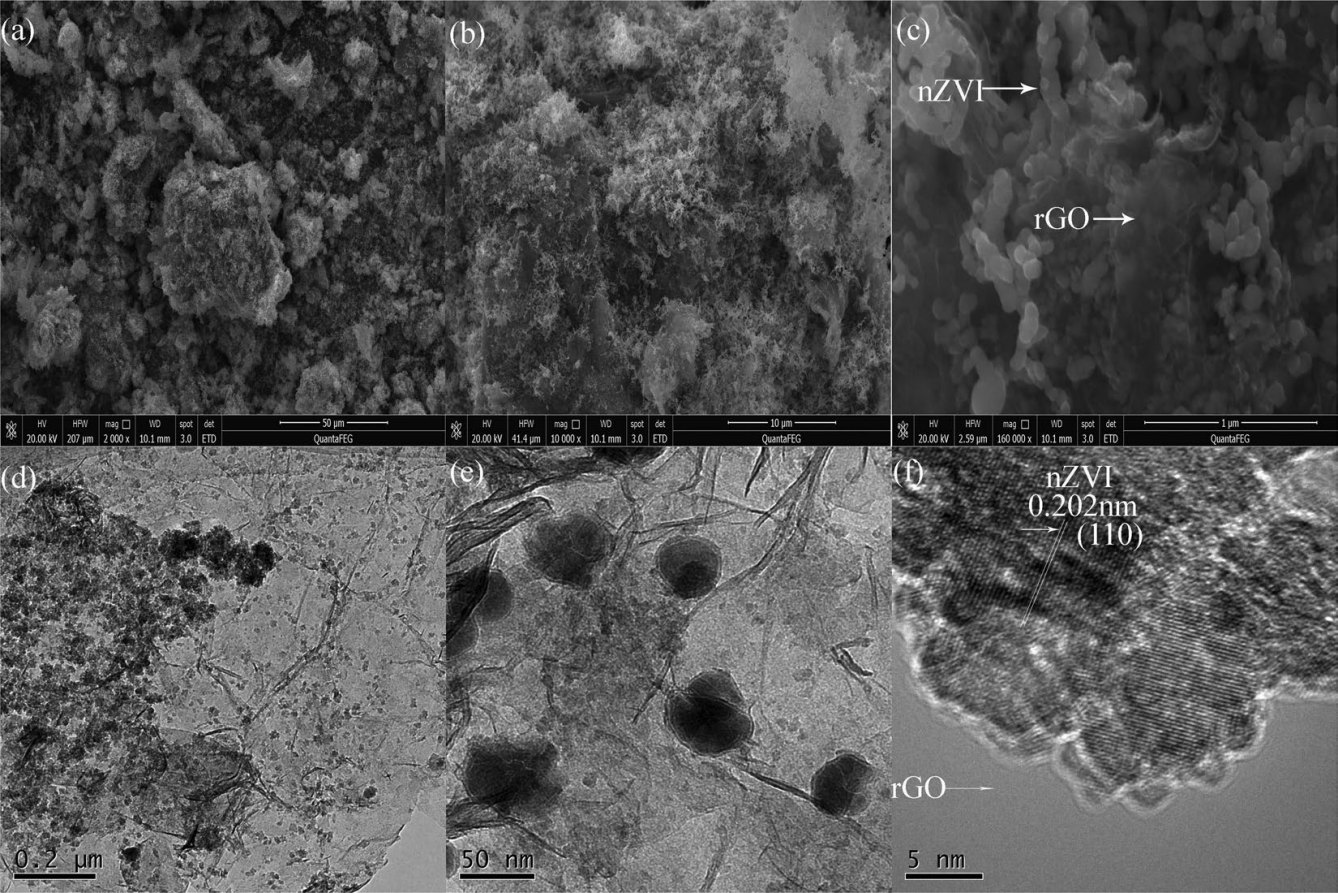
The SEM images of nZVI/rGO magnetic nanocomposites with different magnifications (**a**,**b** and **c**), TEM images of nZVI/rGO magnetic nanocomposites with different magnifications (**d** and **e**), HR-TEM image of nZVI/rGO magnetic nanocomposites (**f**).

As can be seen in Fig. 1a and their corresponding magnified images (Fig. 1b and Fig. 1c), the nZVI particles were successfully dispersed on the surface of rGO to form the nZVI/rGO magnetic nanocomposites. The reason for this is that graphene as a new synthetic 2D allotrope of carbon possesses a high surface area and chemical stability^33^. The average size of nZVI particles on rGO estimated from Fig. 1c was about 40 nm (Fig. S1). Moreover, the rGO sheets showing the folding nature are clearly visible, which indicated that the rGO sheets were of multiple layers (Fig. 1d,e). As shown in Fig. 1f, the nZVI/rGO magnetic nanocomposites present a crystalline lattice spacing (0.202 nm), which corresponds to the (110) lattice plane of nZVI^33^.

**Figure 2:**
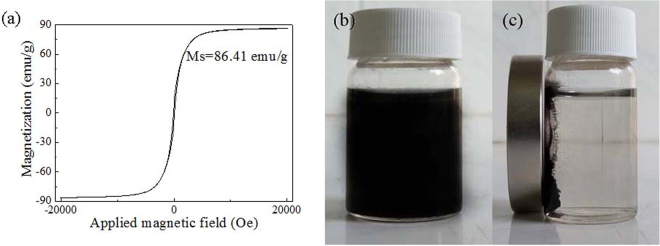
Magnetic hysteresis curve of nZVI/rGO magnetic nanocomposites (**a**), digital photographs showing a water dispersion of nZVI/rGO magnetic nanocomposites (**b**) and the magnetic separation of nZVI/rGO nanocomposites after 10 seconds (**c**).

The magnetization property of the nZVI/rGO nanocomposites was explored at 298 K by evaluating the magnetic hysteresis curve (Fig. 2a). The saturation magnetization of the nZVI/rGO nanocomposites was 86.41 emu/g, which was sufficient for magnetic separation with a conventional magnet. The nZVI/rGO magnetic nanocomposites dispersed in an aqueous solution can be separated within 10 seconds by a magnet, which would make the removal process easier and save more time or economic costs (Fig. 2b,c).

### ANN modelling

**Figure 3:**
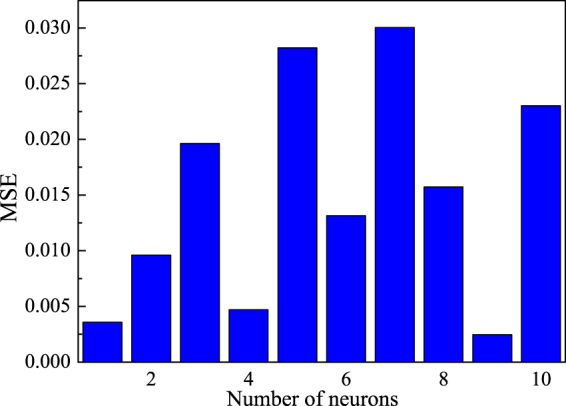
Relationship between MSE and number of neurons in hidden layer.

**Figure 4:**
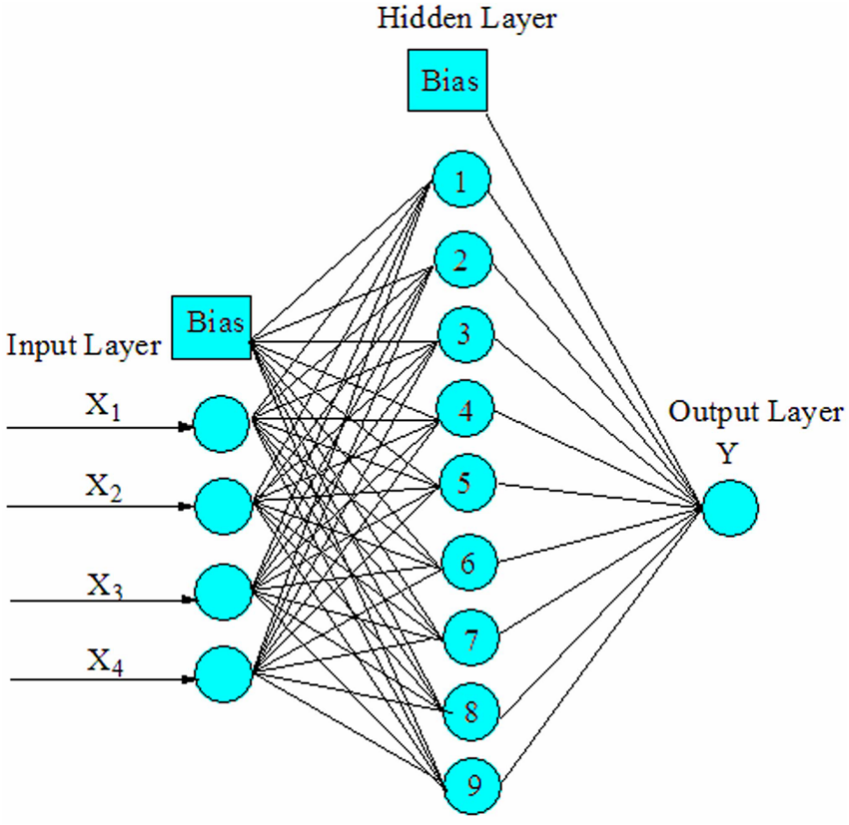
Optimized structure of the BP-ANN.

Although the number of neurons in the hidden layer is in direct proportion with the simulation performance of the ANN model, excessive number of the neurons could lead to over-fitting that could reduce the robustness and generalization of the ANN model^34^. For modeling the Cu(II) removal process, different numbers of neurons (1–10) in hidden layer were used to determine the optimum network architecture based on the relationship between the value of MSE and the number of neurons, thus 9 neurons were selected for the hidden layer (Fig. 3). The optimized BP-ANN with three layers and 9 neurons in the hidden layer is shown in Fig. 4.

**Figure 5:**
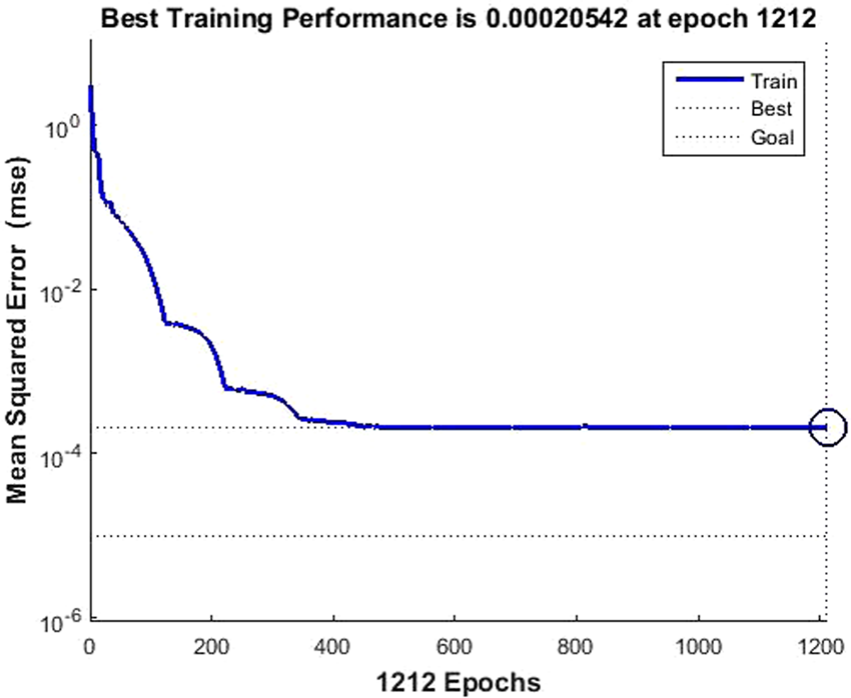
The relationship between MSE and the number of epochs.

**Figure 6:**
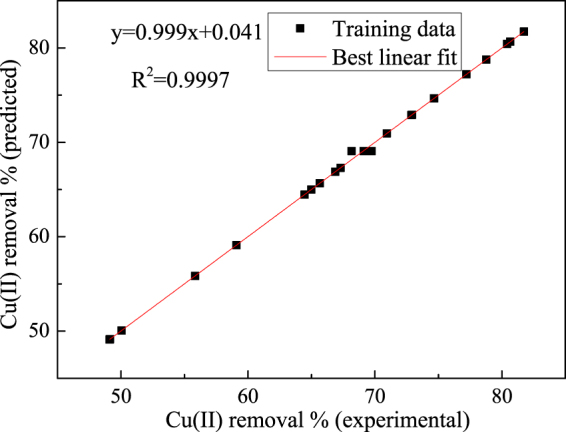
The experimental data versus the predicted data for Cu(II) removal efficiency.

The relationship between the MSE and the number of epochs for the developed BP-ANN for the Cu(II) removal indicated that the training was converged after 1212 epochs with the lowest MSE (0.0002) (Fig. 5). The results predicted by this model indicated that its performance was satisfying due to a low mean absolute error (Table 1). The developed ANN model was validated with the test data generated by RSM. The average absolute error of developed BP-ANN model was 3.64% for the test sets, which demonstrated a good generalization of this model for the Cu(II) removal process with new data. Furthermore, a satisfactory agreement between the experimental and predicted values was obtained with 0.9997 of R^2^ (Fig. 6).

**Table 1:** The relationship between the experimental results and results predicted by the developed BP-ANN. (*represents test sets).

Number of dataset	Operating temperature °C	Initial pH	Initial concentration mg/L	Contact time min	Removal efficiency (%)		Absolute error (%)
					Experimental	Predicted	
1	30	5	200	20	69.7500 ± 0.87	69.0604	0.6896
2	30	6	150	20	74.6500 ± 0.62	74.6505	0.0005
3	40	6	200	20	72.8800 ± 0.96	72.8808	0.0008
4	30	5	200	20	69.1200 ± 1.02	69.0604	0.0596
5	20	5	200	30	72.9200 ± 0.25	72.9201	0.0001
6	30	5	250	30	78.7700 ± 0.38	78.7659	0.0041
7	30	6	200	10	59.1000 ± 0.36	59.0984	0.0016
8	40	5	150	20	74.6600 ± 0.58	74.6596	0.0004
9	30	5	150	10	65.6700 ± 0.95	65.6712	0.0012
10	30	4	250	20	65.0000 ± 0.46	64.9988	0.0012
11	30	4	150	20	77.2000 ± 0.48	77.1986	0.0014
12	40	4	200	20	70.9400 ± 0.69	70.9404	0.0004
13	30	5	150	30	81.7400 ± 0.12	81.7349	0.0051
14	30	4	200	10	64.4600 ± 0.53	64.4597	0.0003
15	30	5	200	20	68.1600 ± 0.59	69.0604	0.9004
16	20	6	200	20	55.8400 ± 0.82	55.8399	0.0001
17	40	5	250	20	66.8800 ± 0.38	66.8795	0.0005
18	30	5	200	20	69.2100 ± 0.61	69.0604	0.1496
19	30	6	200	30	80.4100 ± 0.23	80.4136	0.0036
20	30	6	250	20	67.3000 ± 0.98	67.3005	0.0005
21	30	4	200	30	80.6600 ± 0.79	80.6652	0.0052
22	20	5	250	20	49.1400 ± 0.35	49.1399	0.0001
23	40	5	200	10	50.0600 ± 0.22	50.0597	0.0003
24	30	5	250	10	49.1100 ± 0.60	49.1111	0.0011
25^*^	40	5	200	30	73.7500 ± 0.57	68.6545	5.0956
26^*^	20	5	150	20	64.9900 ± 0.19	69.8168	4.8268
27^*^	20	4	200	20	65.0200 ± 0.88	60.6732	4.3468
28^*^	20	5	200	10	50.1200 ± 0.65	47.3305	2.7895
29^*^	30	5	200	20	67.9200 ± 0.59	69.0604	1.1404
					Mean absolute error		0.6906

The connection weights between neurons in input, hidden and output layers of the developed BP-ANN mode are shown in Table 2, based on which the sensitivity analysis was carried out by using the Garson equation in order to evaluate the relative importance of the input variables on the output variable. As can be seen in Table 3, contact time appears to be the most influential variable followed by initial concentration, operating temperature and initial pH. This is consistent with the results of ANOVA (Table S1).

**Table 2:** Weights and biases in input-hidden layers (W_i_ and b_i_) and hidden-output layers (W_j_ and b_j_).

Number of neurons	W_i_				b_i_	W_j_	b_j_
	Input variables						
	Operating temperature	Initial pH	Initial concentration	Contact time			
1	1.1405	0.1820	−0.9163	−1.8877	−2.7333	−0.5250	
2	−0.7464	−0.1009	1.3385	−1.9665	1.1898	−0.1374	
3	−1.8672	−0.7593	1.0404	1.0547	1.2507	0.0305	−0.3470
4	−1.0621	1.3521	−1.0109	−0.0096	1.3992	0.2202	
5	−0.4985	−0.3383	−0.6612	−0.4575	−0.1313	0.7031	
6	0.3846	−0.2592	−1.4244	−1.6311	0.6935	−0.6361	
7	−0.4123	−0.9827	0.7692	1.9676	−1.0993	0.1897	
8	2.1261	0.5988	−0.4158	1.2984	1.3765	0.3528	
9	2.4359	−0.6441	−1.1226	2.6906	1.8163	0.6953	
10	0.4832	−0.2556	0.8146	0.5108	2.7942	−0.4049

**Table 3:** Relative importance of input variables on the output.

Input variables	Relative importance (%)	Order
Operating temperature	26.28	3
Initial pH	12.64	4
Initial concentration	27.12	2
Contact time	33.96	1

Another approach for sensitivity analysis was also used to determine the influence of a variable in the developed ANN model. Moreover, the performances of interaction of different variables were also investigated. Performances for the groups of one, two, three and four variables were evaluated by the optimal ANN model using the traingdx with 9 hidden neurons. The results showed that contact time was the most effective variable among all variables in the group of one variable because of the low MSE (0.15939) (Table 4). The MSE decreased from 0.15939 to 0.05350 that is the minimum value for the group of two variables (X_1_ + X_4_). The minimum value of MSE in the group of three variables was 0.00368 by the combination of X_1_, X_3_ and X_4_. Then, the MSE decreased down to 0.00020 that is the minimum value for the group of four variables (X_1_ + X_2_ + X_3_ + X_4_). Therefore, it can be concluded that contact time has the most influence on the Cu(II) removal, which is in accordance with the results of the Garson equation.

**Table 4:** The performance evaluation of possible combinations of input variables.

Combination	MSE	Epoch	Correlation coefficient (R^2^)	Best linear equation
X_1_	0.28934	472	0.3718	y = 0.14x + 0.140
X_2_	0.32093	342	0.2100	y = 0.044x + 0.150
X_3_	0.27335	295	0.4311	y = 0.19x + 0.130
X_4_	0.15939	295	0.7247	y = 0.53x + 0.077
X_1_ + X_2_	0.27050	397	0.4408	y = 0.19x + 0.130
X_1_ + X_3_	0.22340	262	0.5785	y = 0.33x + 0.110
X_1_ + X_4_	0.05350	234	0.9169	y = 0.84x + 0.026
X_2_ + X_3_	0.25790	1992	0.4815	y = 0.23x + 0.130
X_2_ + X_4_	0.14072	271	0.7622	y = 0.58x + 0.068
X_3_ + X_4_	0.08787	556	0.8592	y = 0.74x + 0.042
X_1_ + X_2_ + X_3_	0.14535	646	0.7530	y = 0.57x + 0.070
X_1_ + X_2_ + X_4_	0.04298	1372	0.9338	y = 0.87x + 0.021
X_1_ + X_3_ + X_4_	0.00368	1987	0.9945	y = 0.99x + 0.0019
X_2_ + X_3_ + X_4_	0.04756	1999	0.9265	y = 0.86x + 0.023
X_1_ + X_2_ + X_3_ + X_4_	0.00020	1212	0.9997	y = 0.99x + 0.041

### Optimization by GA technique

The developed ANN model was then optimized by GA approach with the objective of the maximization of Cu(II) removal efficiency from aqueous solutions. This approach began with a population of random regimes using temperature, initial pH, initial concentration and contact time as the optimization parameters. As shown in Fig. S2, the value of removal efficiency reached to a maximum value (84.78%) and then remained constant after about 14 generations. The optimized conditions of the four variables were found to be 38.90 °C, 4.67, 155.10 mg/L and 28.90 min. Verification experiments indicated that 83.65% ± 0.62 of Cu(II) removal efficiency is reasonably close to the predicted value (84.78%), which demonstrated the adequacy of the ANN-GA model.

### ANN-PSO optimization

The PSO technique was hybridized with the developed ANN model for optimizing the process parameters with the aim of maximizing the Cu(II) removal efficiency. The relationship between the removal efficiency and iterations demonstrated that after 8 iterations the removal efficiency reaches to the maximum value and then remains constant. The optimal conditions for the Cu(II) removal process were found to be: 20.18 °C, 5.79, 150 mg/L and 30 min. The Cu(II) removal efficiency achieved under the optimal conditions was 86.80 ± 0.72%, which was compatible with the hybrid ANN-PSO prediction (87.26%) (Fig. S3). The absolute error (0.46%) between the experimental and predicted value proved that the model of PSO combined with ANN is an efficient and effective tool for the Cu(II) removal process.

### Comparison among RSM, ANN-GA and ANN-PSO models

The comparison among the RSM, ANN-GA and ANN-PSO models demonstrates a better accuracy of ANN-based models with the higher R^2^ value than that of the RSM model (Table 5). In addition, the absolute error of verification experiments by ANN-PSO model was lower than that of the ANN-GA model (1.13%) and the RSM model (7.44%). Using the ANN-PSO model, the efficiency of Cu(II) removal from aqueous solutions was improved by 3.15% and 8.54% in comparison with the ANN-GA model and the RSM model. Therefore, it can be deduced that although RSM is a widely employed approach for the optimization of Cu(II) removal process, ANN-PSO methodology may present a satisfying alternative.

**Table 5:** The optimized process parameters for Cu(II) removal by nZVI/rGO magnetic nanocomposites using different approaches.

Process parameters	RSM		ANN-GA		ANN-PSO	
	Optimized values	Experimental values	Optimized values	Experimental values	Optimized values	Experimental values
Operating temperature (°C)	39.26	39.30	38.90	38.90	20.18	20.10
Initial pH	6.00	6.00	4.67	4.70	5.79	5.80
Initial Cu(II) concentration (mg/L)	250.00	250.00	155.10	155.00	150.00	150.00
Contact time (min)	30.00	30.00	28.90	28.90	30.00	30.00
Removal efficiency (%)	85.7	78.26 ± 0.57%	84.78	83.65 ± 0.62	87.26	86.80 ± 0.72%
Average values of absolute errors (%)	7.44		1.13		0.46	
R^2^	0.9572		0.9997			

### Removal kinetics

**Figure 7:**
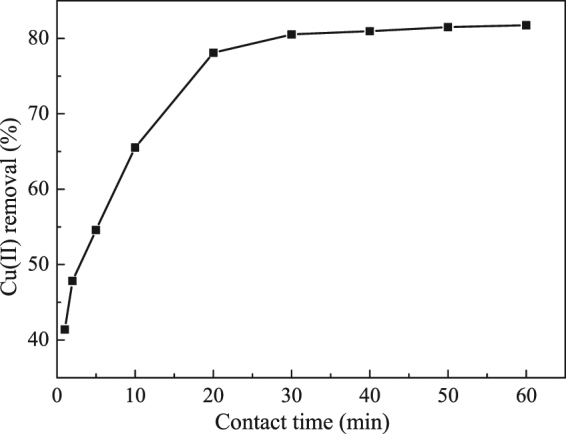
Time-dependent Cu(II) removal by the nZVI/rGO magnetic nanocomposites. (initial pH = 6.00, temperature = 20 °C, composites dosage = 30 mg, and initial concentration = 100 mg/L).

The effect of contact time on the Cu(II) removal by the nZVI/rGO magnetic nanocomposites showed that the removal of Cu(II) is rapid in the first 20 min of contact time and then remains constant with a rise in contact time (Fig. 7). The shaking time (1 h) was selected to ascertain the removal equilibrium of Cu(II) by the nZVI/rGO magnetic nanocomposites. In general, the removal process is rapid and hence 30 min is enough to obtain the removal equilibrium. This is a critical advantage for the application of the nZVI/rGO magnetic nanocomposites to remove heavy metals from aqueous solutions in practical applications.

In order to quantitatively express the Cu(II) removal capacity by nZVI/rGO magnetic nanocomposites, the experimental data at various contact times for the Cu(II) removal were fitted to four kinetic models, such as pseudo-first order, pseudo-second order, intraparticle diffusion and Elovich models (the details of these kinetic models are described in the supplementary information)^35^. It was found that the value of calculated q_e_ for the pseudo-first-order kinetics (79.56 mg/g) is lower than the experimental q_e_ (136.25 mg/g) with the low R^2^ (0.9873), while the value of calculated q_e_ (138.89 mg/g) agrees with the experimental q_e_ in the case of the pseudo-second-order kinetic model (Table 6). Furthermore, the experimental data are also well fitted to intraparticle diffusion model with a higher value of R^2^ (0.9962) than that of the pseudo-first-order kinetic model (R^2^ = 0.9873), the pseudo-second-order kinetic model (R^2^ = 0.9901) and the Elovich model (R^2^ = 0.9640). These results showed that the removal behavior may be described by the pseudo-second-order kinetic model combined with the intraparticle diffusion model, which indicated that the intraparticle diffusion resistance of Cu(II) inside the nZVI/rGO magnetic nanocomposites can influence the removal rate. In summary, the rapid adsorption of Cu(II) onto the external surface of nZVI/rGO magnetic nanocomposites is followed by slow intraparticle diffusion along the mesoporous walls.

**Table 6:** Removal kinetic parameters for Cu(II) by the nZVI/rGO magnetic nanocomposites.

Model	Parameters	Values of parameters	Experimental q_e_ (mg/g)
Pseudo-first-order kinetics	k_1_ (1/min)	0.1244	
	q_e_ (mg/g)	79.56	
	R^2^	0.9873	136.25
Pseudo-second-order kinetics	k_2_ (g/mg/min)	0.0040	
	q_e_ (mg/g)	138.89	
	R^2^	0.9901	
Intraparticle diffusion		17.38	
	k_3_(mg/g/min^1/2^)		
	B (mg/g)	53.19	
	R^2^	0.9962	
Elovich equation	α (mg/g/min)	544.41	
	β (g/mg)	0.05	
	R^2^	0.9640	

### Thermodynamic study

It is hypothesized that energy cannot be gained or lost in an isolated system and the entropy change is the only driving force based on fundamental thermodynamic concepts^36^. Therefore, estimate of thermodynamic parameters has a great significance for evaluating the spontaneity and feasibility of the removal processes. In order to investigate the influence of temperature on the removal of Cu(II) by nZVI/rGO magnetic nanocomposites, batch removal experiments were carried out at different operating temperatures varying from 293 to 323 K. The thermodynamic parameters (Gibbs free energy (ΔG°), enthalpy change (ΔH°) and entropy change (ΔS°)) of the removal process were calculated by the Van’t Hoff equation:

1 $$\mathrm{ln}\,{K}_{d}=\frac{{\rm{\Delta }}{S}^{0}}{R}-\frac{{\rm{\Delta }}{H}^{0}}{RT}$$

2 $${\rm{\Delta }}{G}^{0}=-RT\,\mathrm{ln}\,{K}_{d}$$

where *K*_*d*_ stands for the equilibrium constant, *R* is the gas constant (8.314 J/mol/K), *T* is the absolute temperature (K), *C*_*e*_ and *q*_*e*_ are the equilibrium concentration (mg/L) and adsorption capacity (mg/g) of heavy metal ions, respectively. The value of *K*_*d*_ can be determined by plotting ln(*q*_*e*_/*C*_*e*_) against *q*_*e*_ and extrapolating the linear plot to zero *q*_*e*_^37^.

The values of ΔH° and ΔS° were determined from the slope and intercept of Fig. S4 and are summarized in Table 7. The K_d_ values increased with a rise in the temperature indicating an enhancement interaction between the adsorbate and adsorbent at higher temperatures. The positive value of ΔH° indicated that the removal process was endothermic demonstrating that this process consumes energy. Furthermore, the positive value of ΔS° represented the randomness nature of the Cu(II) removal process at the solid/solution interface. The negative ΔG° values (−26.9590 to −13.1261 kJ/mol) suggested that the Cu(II) removal process was spontaneous in nature and the mechanism for this process was physisorption.

**Table 7:** Thermodynamic parameters of Cu(II) removal by nZVI/rGO magnetic nanocomposites.

ΔS° (kJ/mol/K)	ΔH° (kJ/mol)	ΔG° (kJ/mol)			
0.4663	123.9997	293 K	303 K	313 K	323 K
		−13.1261	−16.5417	−21.8946	−26.9590

### Adsorption isotherms

The adsorption process proceeded until the adsorbent and adsorbate achieve a dynamic equilibrium^38^. In order to evaluate the relationship between Cu(II) and the nZVI/rGO magnetic nanocomposites, the experimental data for Cu(II) removal were fitted to several isotherms, such as Langmuir, Frenudlich, Dubinin-Radushkevich (D-R) and Temkin isotherms (the more detailed descriptions for the four isotherms are presented in the supplementary information). Furthermore, statistical analysis was carried out to determine the validity of isotherms on the basis of four parameters, such as R^2^, chi square test (*x* ^2^), average percentage errors (APE), the sum of absolute errors (SAE)^39^.

The amount of Cu(II) ions adsorbed on the nZVI/rGO nanocomposites increased with a rise in initial Cu(II) concentration (Fig. S5), since high concentrations provided a driving force for the ion transportation from the solution to the nZVI/rGO nanocomposites. As can be seen in Table 8 and Fig. S6, the high value of R^2^ and the low values of *x* ^2^, APE and SAE were obtained for the Langmuir isotherm. This fact demonstrated that the Cu(II) ions form homogeneous monolayer coverage on the surface of nZVI/rGO magnetic nanocomposites. The maximum adsorption capacity was calculated to be 476.19 mg/g, which was in satisfactory agreement with the experimental value (433.88 mg/g). As given in Table 9, the removal capacity of the nZVI/rGO magnetic nanocomposites is significantly higher than that of other materials. The excellent Cu(II) removal capacity and magnetic separation ability of nZVI/rGO nanocomposites are the important advantages for the environmental remediation. The adsorption capacity of Cu(II) by the nZVI/rGO composites was lower than that of Pb(II) (904 mg/g) and was higher than that of Cd(II) (46.45 mg/g) determined in our earlier work^40,41^. The reason for this is that the values for covalent index, atomic weight, electronegativity and ionic radius of Pb(II) were higher than those of Cu(II) and Cd(II) (Table 10). In addition, the values of R_L_ in this work vary from 0.05 to 0.40 indicating that the adsorption of Cu(II) ions by nZVI/rGO magnetic nanocomposites is more favorable at higher initial concentrations than at lower initial concentrations (Fig. S7).

**Table 8:** Isotherm parameters for the adsorption of Cu(II) onto the nZVI/rGO magnetic nanocomposites.

Isotherms	Langmuir		Freundlich		Temkin		Dubinin-Radushkevich	
Constants	K_L_ (L/mg)	0.03	K_F_ (mg/g)	65.37	RT/a_t_ (kJ/mol)	0.09	q_m_ (mg/g)	371.34
	q_max_ (mg/g)	476.19	n	2.93	b_t_ (L/g)	0.37	α (mol^2^/J^2^)	3 × 10^−5^
	R^2^	0.9961	1/n	0.34	R^2^	0.9845	E (kJ/mol)	0.37
			R^2^	0.9736			R^2^	0.8282
*x* ^2^	3.58		8.23		10.11		50.24	
APE	0.03		0.05		0.06		0.15	
SAE	67.97		106.96		123.73		293.90	
Experimental q_max_ (mg/g)	433.88

**Table 9:** Comparison of Cu(II) removal capacity by the nZVI/rGO magnetic nanocomposites with other materials.

Materials	q_max_ (mg/g)	Reference
Cystoseira crinitophylla biomass	160.00	42
Fe_3_O_4_@SiO_2_-EDTA	37.59	4
GO/SiO_2_	158.90	43
Magnetic chitosan	35.50	45
PAA/PVA	49.30	46
PAH-GO	349.03	15
Carbon based adsorbent	33.33	47
Bentonites	43.10	48
EDTA-MNP	73.26	49
nZVI/rGO magnetic nanocomposites	476.19	Present study

**Table 10:** The physical properties of Pb(II), Cu(II) and Cd(II).

	Pb(II)	Cu(II)	Cd(II)
Covalent index	5.559	4.874	4.278
Atomic weight	207.2	63.54	112.411
Electronegativity	1.9	1.9	1.7
Ionic radius	1.19	0.73	0.97

The value of K_F_ for the Freunlich model was 38.11 illustrating that the nZVI/rGO magnetic nanocomposites possessed an excellent adsorption capacity for Cu(II), which was higher than that of other materials^8,42,43^. In addition, the values of n and 1/n were found to be 2.93 and 0.34 demonstrating that the Cu(II) adsorption onto the nZVI/rGO magnetic nanocomposites was favorable under the studied conditions. The low value of RT/a_T_ (0.09 kJ/mol) for the Temkin isotherm indicated that this adsorption process is dominated by a physical adsorption. It is known that the adsorption took place by ion exchange when the adsorption mean free energy (E) for the D-R model lied between 8 and 16 kJ/mol, whereas the adsorption proceeded physically when the value of E was below 8 kJ/mol^44^. The value of E in this study was found to be 0.37 kJ/mol, therefore the adsorption of Cu(II) onto nZVI/rGO magnetic nanocomposites is of a physical type^45-49^.

### Removal mechanism of Cu(II)

**Figure 8:**
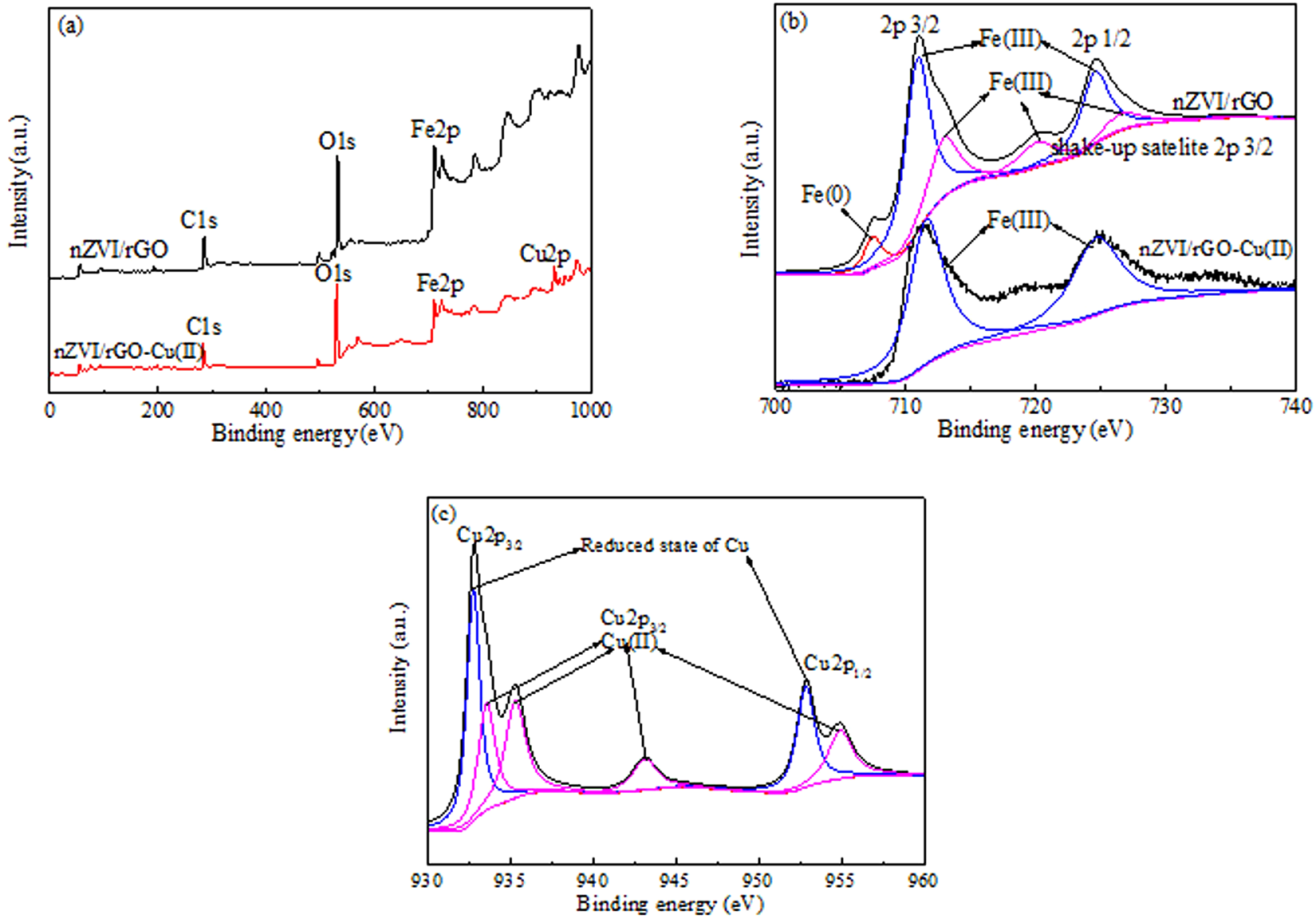
XPS analysis of nZVI/rGO composites reacting with Cu(II): wide scan of nZVI/rGO and nZVI/rGO-Cu(II) nanocomposites (**a**); Fe 2p of nZVI/rGO and nZVI/rGO-Cu(II) nanocomposites **(b)**; Cu 2p of nZVI/rGO-Cu(II) nanocomposites (**c**). (100 mg/L Cu(II) solution, initial pH = 6.00, composites dosage = 30 mg, contact time = 1 h and temperature = 20 °C).

The heavy metal ions removal from aqueous solutions by nZVI/rGO magnetic nanocomposites is dominated by various mechanisms, such as complex formation, electrostatic interaction, ion-exchange, precipitation and reduction^33,50^. The XPS spectra of wide scan, Fe 2p and Cu 2p for the nanocomposites before and after the Cu(II) removal were measured to investigate the removal mechanisms by the nZVI/rGO magnetic nanocomposites (Fig. 8). The wide scan spectrum of nZVI/rGO indicated the existence of carbon, oxygen and iron, while the peak of Cu appeared in the wide scan of nZVI/rGO-Cu(II). The presence of this peak demonstrated the Cu(II) immobilization on the surface of nZVI/rGO (Fig. 8a). The Fe 2p spectrum of nZVI/rGO contains four peak at 706.80 eV, 711 eV, 720 eV and 725 eV, representing the binding energies of 2p 3/2, shake-up satellite 2p 3/2 and 2p 1/2, respectively (Fig. 8b). This result indicated the existence of a layer of iron oxides, which was ascribed to Fe_2_O_3_ + FeO. In addition, a small peak at 706.80 eV demonstrates the presence of zero-valent iron (Fe^0^). The relative intensity of Fe^0^ was significantly lower than that of Fe(II) and Fe(III), which revealed the core-shell structure of nZVI. After the Cu(II) removal, the Fe^0^ and Fe(II) peaks disappeared indicating that Fe^0^ and Fe(II) on the surface of nZVI/rGO were transformed to Fe(III). The Cu 2p spectrum of nZVI/rGO-Cu(II) showed three peaks at 933.10 eV, 943.20 eV and 953.10 eV, representing the energies of 2p 3/2, shake-up satellite 2p 3/2 and 2p 1/2 (Fig. 8c). Two peaks at 932.75 eV and 952.85 eV illustrated that the reduction of Cu(II) also took place on the surface of the nZVI/rGO magnetic nanocomposites. This result was in good agreement with the analysis of Fe 2p spectrum.

In our earlier studies, we found that Cd(II) ions cannot be reduced to Cd(0) and a small part of Pb(II) ions was reduced to Pb(0) by the nZVI/rGO nanocomposites^40,41^. The metal ions with a significantly more positive standard reduction potential (STP) than that of Fe^0^ can be removed by a reduction mechanism, while the metals having a STP more negative than or close to that of Fe^0^ can be removed by an adsorption mechanism. The order for the standard reduction potential (STP) of these heavy metals was: Cu(II) (0.34 V) >Pb(II) (−0.12 V) >Cd(II) (−0.35 V) >Fe(II) (−0.44 V). Therefore, the Cu(II) removal mechanism by nZVI/rGO nanocomposites was controlled by the adsorption and reduction.

## Conclusions

In this study, the nZVI/rGO magnetic nanocomposites were prepared and applied to remove the Cu(II) ions from aqueous solutions. The characterizations of nZVI/rGO nanocomposites were performed by using TEM, SQID magnetometer and XPS, showing the morphology, magnetic and surface properties. The process parameters (operating temperature, initial pH, initial concentration and contact time) of the Cu(II) removal by nZVI/rGO nanocomposites were optimized by RSM, ANN-GA and ANN-PSO. The results showed that the ANN model offered more accurate predictions than the RSM model with a higher R^2^ value and a lower MSE value. Both ANOVA and sensitivity analysis demonstrated that the most critical parameter was contact time for the Cu(II) removal. Using the ANN-PSO based tool, the Cu(II) removal efficiency from aqueous solutions was improved by 3.15% and 8.54% as compared to that of the ANN-GA model and the RSM model. The high removal efficiency and low absolute error of the ANN-PSO model indicated that this model was proven to be an alternative for modeling and optimizing the Cu(II) removal process. Furthermore, experimental data were best fitted to the Langmuir isotherm with 476.19 mg/g of maximum adsorption capacity. The removal kinetics of Cu(II) by the nZVI/rGO magnetic nanocomposites followed the pseudo-second order kinetic model and intraparticle diffusion model. The thermodynamic parameters revealed that the Cu(II) removal process is spontaneous and endothermic in nature. Finally, the Cu(II) removal by nZVI/rGO magnetic nanocomposites was investigated by the XPS analysis, which demonstrated that the removal process was controlled by the adsorption and reduction mechanisms. Therefore, the nZVI/rGO magnetic nanocomposites is suitable for the remediation of Cu(II) pollution because of its high removal efficiency and easy magnetic separation. Future studies can be carried out concerning the modeling and optimization of the complex removal processes with the aid of empirical models (e.g., RSM and ANN models) and mechanistic models combined with more advanced AI techniques.

## Materials and Methods

### Materials

In this study, graphite powder (<30 µm) was supplied by Sinopharm Chemical Reagent. All chemicals of analytical grades (CuSO_4_·5H_2_O, FeSO_4_·7H_2_O, NaOH and HCl) were used without further purification. The Cu(II) stock solution (1000 mg/L) was prepared by dissolving an amount of CuSO_4_·5H_2_O in deionized water, which was further diluted for a desired concentration. The preparation procedure of nZVI/rGO magnetic nanocomposites was referred to the supplementary information.

### Characterization of nZVI/rGO magnetic nanocomposites

The size and morphology of nZVI/rGO nanocomposites were measured using SEM (Quanta FEG250, FEI, USA) and the high-resolution TEM images of nZVI/rGO nanocomposites were taken by a TecnaiG2 F20 microscope (FEI, USA). The magnetic hysteresis loops of nZVI/rGO nanocomposites were recorded on a SQUID magnetometer (MPMS XL-7, Quantum Design, USA). The XPS measurements were recorded on an ESCALAB 250Xi spectrometer (Thermo Fisher Scientific, USA) with monochromatized Al Kα radiation (1486.6 eV) and all binding energies were corrected with the binding energy of C1s as a reference. The other characterizations (XRD, SEM, Raman, N_2_ sorption and FTIR) of nZVI/rGO nanocomposites were carried out in our earlier study^40,41^.

### Removal experiments

The Cu(II) removal from aqueous solutions by nZVI/rGO magnetic nanocomposites were carried out in 100 mL centrifuge tubes. A known amount of nZVI/rGO magnetic nanocomposites (30 mg) was added to 50 mL of Cu(II) solutions with various concentrations (50–600 mg/L). The ranges of operating temperature, initial pH and contact time were from 20 to 50 °C, 1 to 6 and 1 to 60 min, respectively. The initial pH of Cu(II) solution was adjusted to the required values by using 0.1 M NaOH or 0.1 M HCl. The nZVI/rGO magnetic nanocomposites were separated from aqueous solutions by a magnet after removal experiments and the residual concentration of Cu(II) was then measured by flame atomic absorption spectrophotometer (WFX-210, Ray Leigh Corporation, Beijing, China). All experiments were performed in triplicate and the average values of the results were used for data analysis.

### Back propagation artificial neural network (BP-ANN)

ANNs are known for their learning, modeling and prediction capacities of the data, which are composed of input, hidden and output layers. The neurons in three layers of an ANN model are the processing units operating independently of others and describe the relationship between independent and dependent variables^51^. Back propagation is an iterative optimization process in which the mean square error is minimized by adjusting the values of the weight and bias between the neurons. The activation functions were used to produce an output by converting a weighted sum of the input^52^. The activation function between the input and hidden layer was a tangent sigmoid transfer function, while the function between the hidden and output layer was a linear transfer function. The number of neurons in hidden layer was chosen based on the minimum value of MSE^53^. The training data for the ANN model were normalized between −1 and 1 to avoid numerical overflows due to large or small weights. The normalized equation can be described as follows:

3 $${y}_{i}=2\frac{x-{x}_{{\rm{\min }}}}{{x}_{{\rm{\max }}}-{x}_{{\rm{\min }}}}-1$$

where *y*_*i*_ stands for the normalized value of *x*, and *x*_*min*_ and *x*_*max*_ represent the minimum and maximum values of *x*, respectively. To compute the weight of a neuron in hidden layer, Eq. (4) can be put forward:

4 $${W}_{b}=\sum _{a=1}^{k}{w}_{ab}\,{x}_{a}$$

where *k* is the number of neuron in input layer, *w*_*ab*_ is the connection weight between neuron *a* in input layer and neuron *b* in hidden layer, and *x*_*a*_ is the value of neuron *a* in input layer. Similarly, the weight of a neuron in output layer can be calculated as follows:

5 $${W}_{c}=\sum _{b=1}^{z}{w}_{bc}\,{x}_{b}$$

where *z* is the number of neuron in hidden layer, *w*_*bc*_ represents the connection weight between neuron *b* in hidden layer and neuron *c* in output layer, and *x*_*b*_ stands for the value of neuron *b* in hidden layer. The weight of neuron in hidden layer or output layer was used in the activation function, which produced a predicted output by Eq. (6).

6 $$y=f(W+B)$$

where *y*, *f*, *W* and *B* are the output, activation function, weight and bias in hidden layer or output layer, respectively.

Sensitivity analysis was carried out to investigate the connection weights of the developed ANN model. To assess the relative importance of the dependent variables on the independent variable for the Cu(II) removal, both Garson equation and possible combination of variables were utilized^54,55^. The Garson equation can be given as follows:

7 $${O}_{eg}=\frac{{\sum }_{f=1}^{n}(\frac{|{w}_{ef}|}{{\sum }_{i=1}^{m}|{w}_{if}|}|{w}_{fg}|)}{{\sum }_{e=1}^{m}({\sum }_{f=1}^{n}(\frac{|{w}_{ef}|}{{\sum }_{i=1}^{m}|{w}_{if}|}|{w}_{fg}|))}$$

where *O*_*eg*_ is the relative effect of the *eth* dependent variable on the *gth* independent variable, *w* is the connection weight, *e*, *f* and *g* are the number of neurons in the input layer, hidden layer and output layer, respectively.

In this study, a three-layered BP-ANN was established with a tansig function at hidden layer and a purelin function at output layer. Input layer has four neurons that represent operating temperature (X_1_), initial pH (X_2_), initial concentration (X_3_) and contact time (X_4_), while output layer has one neuron that represents the Cu(II) removal efficiency. Gradient descent back-propagation with momentum and adaptive learning rate (traingdx) with 2000 iteration and the goal of MSE (10^−5^) were employed. Experimental data sets (29 sets) were generated from RSM, which were randomly divided into two groups (24 sets for training and 5 sets for testing). The range of independent variables (Table S2) was chosen from the single factor experiments, which can be found in Fig. S8. The description of RSM can be found in the supplementary information.

### Genetic algorithm

Genetic algorithm is an AI-based stochastic non-linear optimization method through simulating the biological selection and genetic mechanism^28^. This algorithm begins with a population of random solution by using operating temperature, initial pH, initial concentration and contact time as optimization variables. The fitness function was obtained from the developed BP-ANN model, which can be expressed as follows:

8 $$F=Purelin(JW\ast \,\tan \,sig(KW\ast [{x}_{1};{x}_{2};{x}_{3};{x}_{4}]+{b}_{j})+{b}_{k})$$

where *F* is the removal efficiency, *JW* and *b*_*k*_ represent the weight and bias in output layer, and *KW* and *bj* stand for the weight and bias in hidden layer, respectively.

The optimization for the removal process consists of three steps: selection, crossover and mutation. Selection is a operation that chooses outstanding individuals from the present population in order to propagate an excellent offspring^28^. The purpose of crossover and mutation is to interchange the information and genes between the individuals, while mutation randomly selects individuals in the population and changes a few genes of the individuals^56^. Both crossover and mutation are employed to create the new and better individuals from parents^55^. The optimization parameters applied in this study including the number of input neurons, initial population, maximum generation, crossover probability and mutation probability were 4, 20, 100, 0.8 and 0.01.

### Particle swarm optimization

PSO is an evolutionary algorithm proposed by Kennedy and Eberhart, which can avoid trapping in a local minimum since it is not based on gradient descent algorithm^31^. This was inspired by the simulation of the foraging behavior of birds, which searches for the optimization by updating the generations^57^. PSO has a series of operating parameters, such as the initial population, inertia weight and acceleration coefficients (personal learning coefficient = c1 and global learning coefficient = c2)^32^. It starts with the following steps: (i) generation of initial population with random positions and velocities; (ii) assessment of fitness function for each particle. The former value will be replaced when a new position with better fitness value is obtained; (iii) calculation of the new velocity for the particles; (iv) update the position of particle by moving toward maximal objective function; (v) this operation will be converged until the iteration number reach the maximum^58^. In the present study, the swarm size, maximum iteration, c1, c2, minimum inertia weight and maximum inertia weight were 20, 50, 2, 2, 0.3 and 0.9.

## Electronic supplementary material

Modeling and prediction of copper removal from aqueous solutions by nZVI/rGO magnetic nanocomposites using ANN-GA and ANN-PSO
