# Supplementary material for: Modeling and prediction of copper removal from aqueous solutions by nZVI/rGO magnetic nanocomposites using ANN-GA and ANN-PSO
Source: Sci Rep. 2017 Dec 21;7:18040. doi: 10.1038/s41598-017-18223-y (PMC5740166; doi:10.1038/s41598-017-18223-y)
Supplement: Supplementary file 1 — Modeling and prediction of copper removal from aqueous solutions by nZVI/rGO magnetic nanocomposites using ANN-GA and ANN-PSO [file 41598_2017_18223_MOESM1_ESM.pdf]

Supplementary Information

Modeling and prediction of copper removal from aqueous solutions by nZVI/rGO magnetic nanocomposites using ANN-GA and ANN-PSO

Mingyi Fan[a], Jiwei Hu[a,b*], Rensheng Cao[a], Kangning Xiong[b], Xionghui Wei[c]

[a]Guizhou Provincial Key Laboratory for Information Systems of Mountainous Areas and Protection of Ecological Environment, Guizhou Normal University, Guiyang 550001, Guizhou, China

[b]Cultivation Base of Guizhou National Key Laboratory of Mountainous Karst Eco-environment, Guizhou Normal University, Guiyang 550001, Guizhou, China

[c]Department of Applied Chemistry, College of Chemistry and Molecular Engineering, Peking University, Beijing 100871, China

*Corresponding author

E-mail: jiweihu@yahoo.com or jwhu@gznu.edu.cn; phone: +86-851-8670-2710

Reduced graphene oxide-supported nanoscale zero-valent iron preparation details. The preparation of graphene oxide (GO) was carried out using the improved Hummers method, and reduced graphene oxide (rGO) was synthesized by the reduction of GO with the $NaBH_4$ solution[1,2]. The nZVI/rGO composites were prepared by the reduction of $FeSO_4·6H_2O$ and GO with $NaBH_4$ (mass ratio, carbon:iron = 1:2). 1g of GO was dispersed in deionized water (300 mL) by 2 hours ultrasonication. 10g of $FeSO_4.7H_2O$ was added into the GO suspension, which was mixed for 12 hours with magnetic stirring to reach the adsorption equilibrium. $NaBH_4$ solution (5.46 g/50 mL) was added into the above solution at room temperature with magnetic stirring for 30 min. The prepared nZVI/rGO composites were separated from the liquid phase via centrifugation and dried at 50 °C in the vacuum drying oven for 24 hours.

RSM modeling and optimization. RSM is a commonly utilized statistical method for optimizing the process parameters and providing the statistical relationship among the variables. Namely, operating temperature (X_1), initial pH (X_2), initial concentration (X_3) and contact time (X_4) were selected as independent variables, and the Cu(II) removal efficiency (Y) was considered as the dependent variable. The factor levels were coded as -1 (low), 0 (central point) and 1 (high). The number of experiments needed to investigate the optimization of removal process are 81($(3)^4$), which were reduced to 29 by using a Box-Behnken experimental design. The experimental data were fitted to a second-order multiple regression analysis and analysis of variance (ANOVA) to investigate the behavior of the system using the least squares regression methodology. The quadratic model can be represented as below:

$$Y = c_0 + \sum_{i=1}^{m} c_i x_i + \sum_{i=1}^{m}\sum_{j=1}^{m} c_{ij} x_i x_j + \sum_{i=1}^{m} c_{ii} x_i^2 + a \quad (1)$$

where Y stands for the removal efficiencies of Cu(II), c_0 represents the constant coefficient, c_i, c_{ii} and c_{ij} are the coefficient for linear, quadratic and interaction effect, respectively, x_i and x_j are the values of the independent variables; a is the residual error. All experimental ranges and levels of independent variables chosen are collected in Table S2, which are based on the single factor experiments. The single factor experiments were carried out to provide a

reasonable range for the independent variables of response surface experiments.

Fitting of second order polynomial equations and statistical analysis. The quadratic model with evaluated coefficients for the Cu(II) removal is given as follows:

$$Y=216.32+1.59X_1-45.00X_2-0.55X_3-0.81X_4+0.28X_1X_2+0.004X_1X_3+0.01X_1X_4+0.02X_2X_3+0.13X_2X_4+0.006X_3X_4-0.06X_1^2+2.82X_2^2+0.0002X_3^2-0.01X_4^2 \quad (2)$$

The result of ANOVA for the quadratic model is shown in Table S1, which implies that this model was significant with a low probability value with F value of 22.35. There is only a 0.01% chance that a large "Model F-value" could occur due to the noise. Statistical analysis of the data indicated the values of 0.9572 and 0.9144 for R^2 and adjusted R^2. In addition, the initial concentration, contact time and operating temperature are quite significant for the Cu(II) removal except for initial pH. Based on the F-values of independent variables, the order for these parameters influencing the Cu(II) removal process is: contact time (173.53) > initial concentration (44.39) > operating temperature (33.39) > initial pH (1.90). To better study and understand the effects of the independent variables and their interactions on the response, 3D response surface plots are employed (Fig. S9). The response surface plots showed the influence of two variables on the response at the center level of other variables. The nonlinear nature of 3D response surface plots could demonstrate that there are interactions between each of the independent variables and dependent variable. The predicted maximum Cu(II) removal efficiency by using RSM was 85.70%, and the optimized conditions were X_1 = 39.26 °C, X_2 = 6.00, X_3 = 250.00 mg/L and X4 = 30.00 min. The Cu(II) removal obtained experimentally in RSM optimized condition was 78.26±0.57% with 7.44% of the absolute error.

Removal kinetics. The four kinetic models, such as the pseudo-first order, pseudo-second order, intraparticle diffusion and Elovich models, can be described as follows:

$$\log(q_e - q_t) = \log q_e - \frac{k_1 t}{2.303} \quad (3)$$

$$\frac{t}{q_t} = \frac{1}{k_e {q_e}^2} + \frac{t}{q_e} \tag{4}$$

$$q_t = k_3 t^{0.5} + B \tag{5}$$

$$q_t = \frac{1}{\beta}\ln(\alpha\beta) + \frac{1}{\beta}\ln t \tag{6}$$

where q_e and q_t represent the quantity of Cu(II) removal at equilibrium and at time t, k_1 (1/min), k_2 (g/mg/min) and k_3 (mg/g $\min^{0.5}$) stand for the rate constant of pseudo-first-order kinetic model, pseudo-second-order kinetic model and intraparticle diffusion model, B is the intercept that is associated to the boundary layer thickness, α (mg/g/min) represents an initial removal rate and β (g/mg) is a desorption constant. The pseudo-first-order kinetic model describes the rate of removal to be proportional to the number of unoccupied active sites by the solutes, while the pseudo-second-order kinetic model assumes that the removal rate of Cu(II) is proportional with the square of difference between the quantity of Cu(II) remoed with time and the amount of Cu(II) absorbed at equilibrium[3,4]. The intraparticle diffusion model hypothesizes that the solute uptake changes proportionally with $t^{0.5}$ rather than with the contact time t[5]. The Elovich model was generally applied in chemisorption kinetics, which was found in overlapping a broad range of slow adsorption rate[6].

Adsorption isotherms. The Langumuir isotherm assumes that the adsorption sites are monolayer and identically homogeneous adsorption on the outer surface of adsorbent with a finite number of adsorption sites[7], which can be expressed as follows:

$$\frac{C_e}{q_e} = \frac{1}{k_L q_{\max}} + \frac{C_e}{q_{\max}} \tag{7}$$

where k_L represents the Langmuir adsorption equilibrium constant (L/g), q_{max} stands for the maximum adsorption (mg/g). In addition, the essential feature of the Langmuir isotherm can be evaluated by means of R_L, which is given as follows:

$$R_L = \frac{1}{1 + k_L C_0} \tag{8}$$

The Freudlich model is applied to describe the adsorption process onto the heterogeneous adsorption sites with the

different affinities of binding sites on the surface of nZVI/rGO magnetic nanocomposites and with the distinct interactions with the Cu(II) ions[8].

$$\log q_e = \log K_F + \frac{1}{n}\log C_e \tag{9}$$

where K_F and n stand for the Freundlich constants related to adsorption intensity and adsorption capacity, respectively. The 1/n value between 0 and 1 indicated that the adsorption process of heavy metals was favorable. The Temkin isotherm supposed that the heat of adsorption process decreases linearly as the degree of adsorption increases, which can be represented by the following equation:

$$q_e = \frac{RT}{a_t}\ln b_t + \frac{RT}{a_t}\ln C_e \tag{10}$$

where RT/a_t and b_t are the constants related to the heat (J/mol) and binding energy (L/g) of the adsorption process. The experimental data were also fitted to the D-R isotherm to evaluate the nature of the adsorption process as physical or chemical, which can be expressed as follows:

$$\ln q_e = \ln q_{\max} - \alpha\varepsilon^2 \tag{11}$$

$$\varepsilon = RT\ln(1 + \frac{1}{C_e}) \tag{12}$$

$$E = 2\alpha^{-1/2} \tag{13}$$

where α represents the activity coefficient related to adsorption mean free energy (mol^2/J^2), ε stands for the Polanyi potential and E (kJ/mol) is the mean energy of adsorption.

Source	Sum of Squares	df	Mean Square	F-Value	p-Value	Remarks
Model	2358.31	14	2358.31	22.35	<0.0001	significant
X_1	251.63	1	251.63	33.39	<0.0001	
X_2	14.30	1	14.30	1.90	<0.0001	
X_3	334.54	1	334.54	44.39	<0.0001	
X_4	1307.71	1	1307.71	173.53	<0.0001	
X_1X_2	30.91	1	30.91	4.10	<0.0001	
X_1X_3	16.28	1	16.28	2.16	0.0006	

X_1X_4	5.52	1	5.52	0.73	0.0003	
X_2X_3	5.88	1	5.88	0.78	0.0002	
X_2X_4	6.53	1	6.53	0.87	<0.0001	
X_3X_4	41.86	1	41.86	5.55	0.6041	
X_1^2	229.07	1	229.07	30.40	<0.0001	
X_2^2	51.44	1	51.44	6.83	<0.0001	
X_3^2	1.26	1	1.26	0.17	0.0012	
X_4^2	7.53	1	7.53	1.00	<0.0001	
Residual	105.50	14	7.54			
Lack of Fit	103.15	10	10.32	17.54	0.0070	significant
Pure Error	2.35	4	0.59			
Total	2463.81	28				

Table S1. ANOVA analysis and statistical parameters for the quadratic model.

Parameters	Unit	Lower level (-1)	Middle level (0)	Upper level (+1)
Operating temperature	°C	20	30	40
Initial pH		5	6	7
Initial concentration	mg/L	150	200	250
Contact time	min	10	20	30

Table S2. Independent variables and their levels for the RSM modeling and optimization.

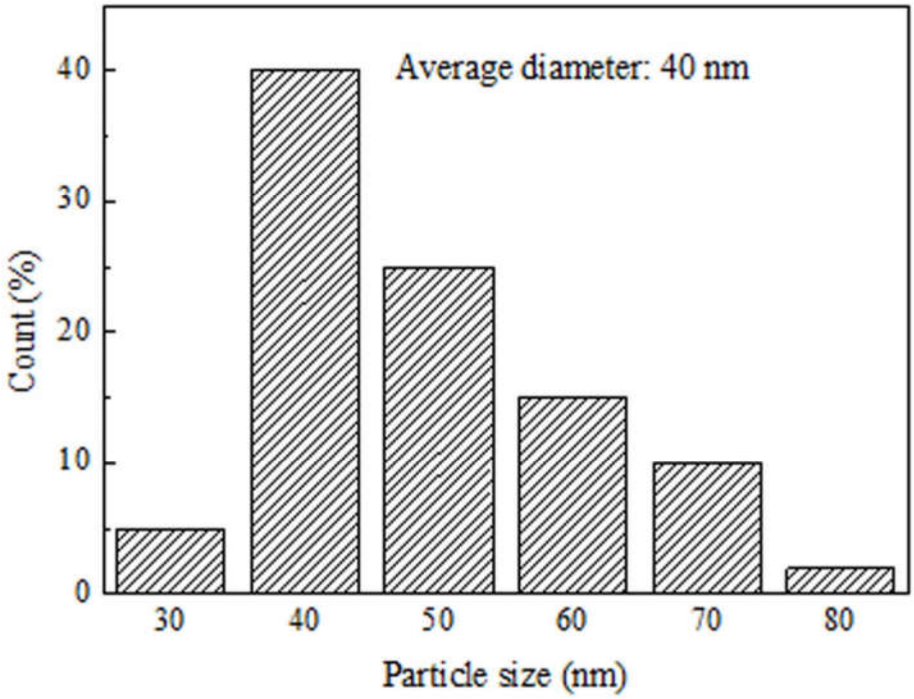

Figure S1. Size distribution calculated from SEM images of nZVI/rGO nanocomposites.

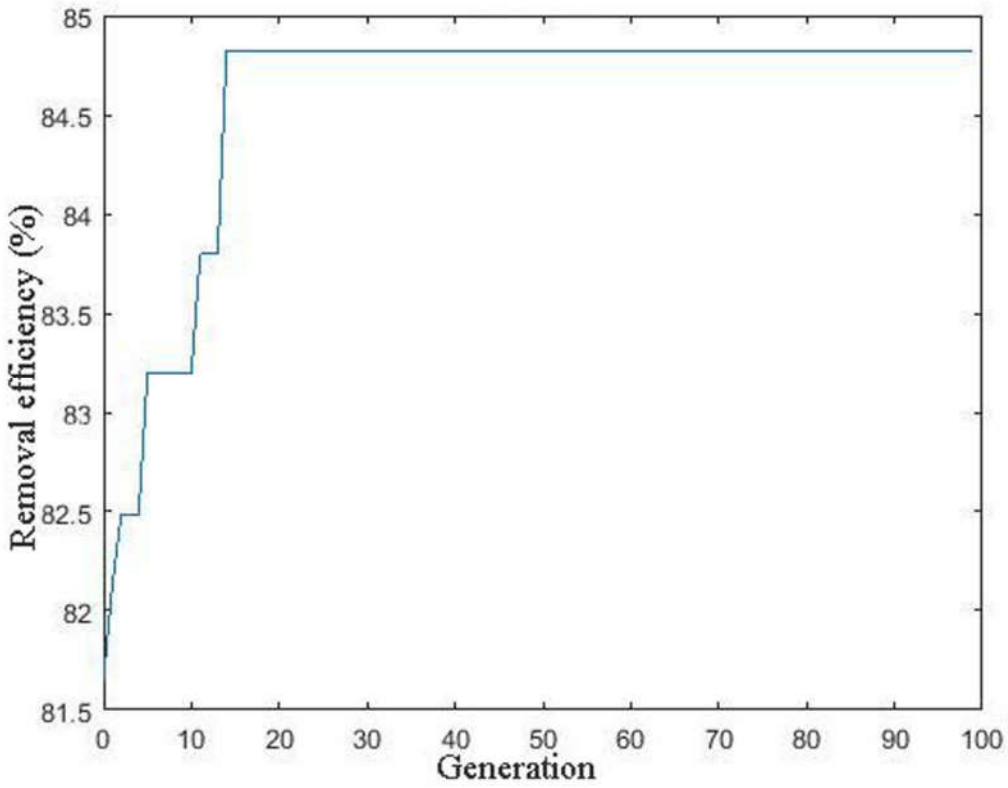

Figure S2. Evolvement of fitness with 100 generations.

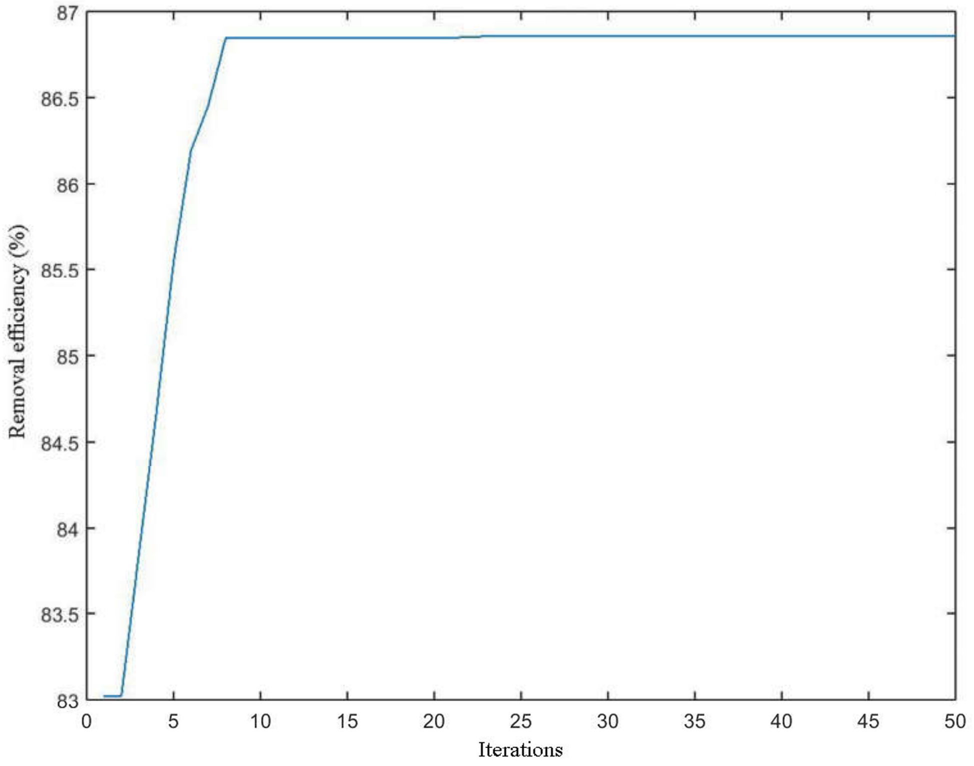

Figure S3. The maximum removal efficiency against iterations.

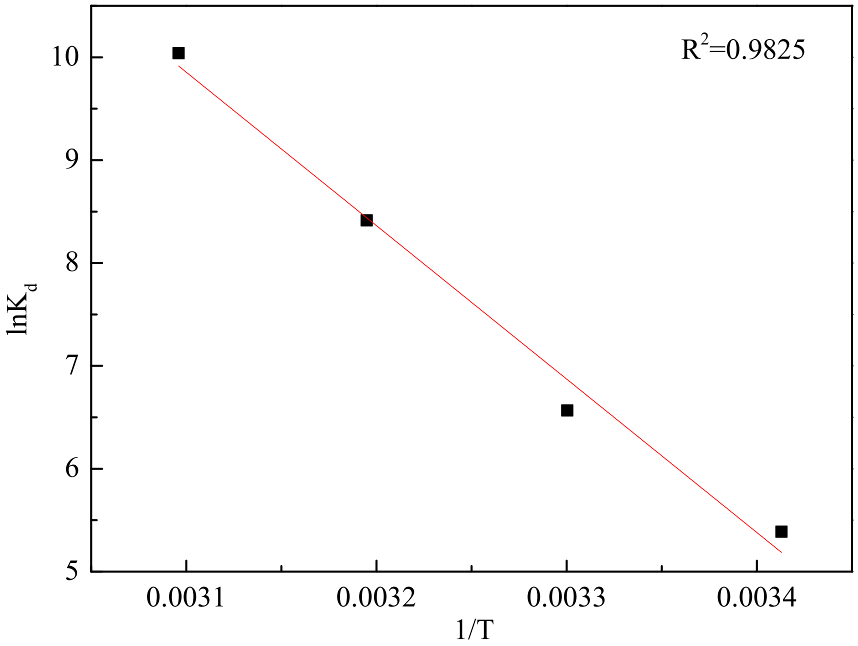

Figure S4. Van't Hoff plot fot the removal of Cu(II) by the nZVI/rGO magnetic nanocomposites. (initial pH = 6.00, composites dosage = 30 mg, initial concentration = 100 mg/L and contact time = 1 h)

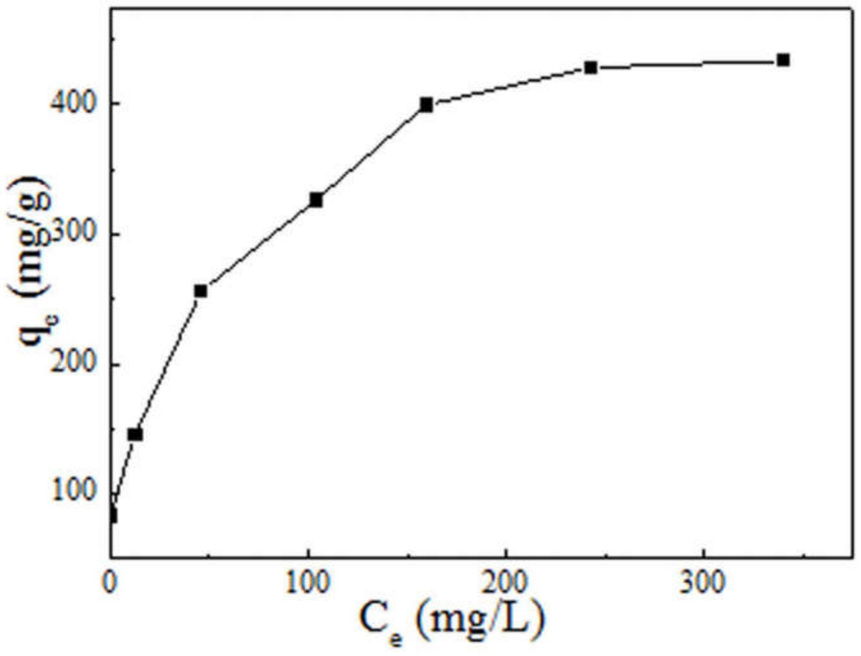

Figure S5. Adsorption isotherm for the adsorption of Cu(II) on the nZVI/rGO magnetic nanocomposites. (initial pH = 6.00, composites dosage = 30 mg, contact time = 1 h and temperature = 20 °C)

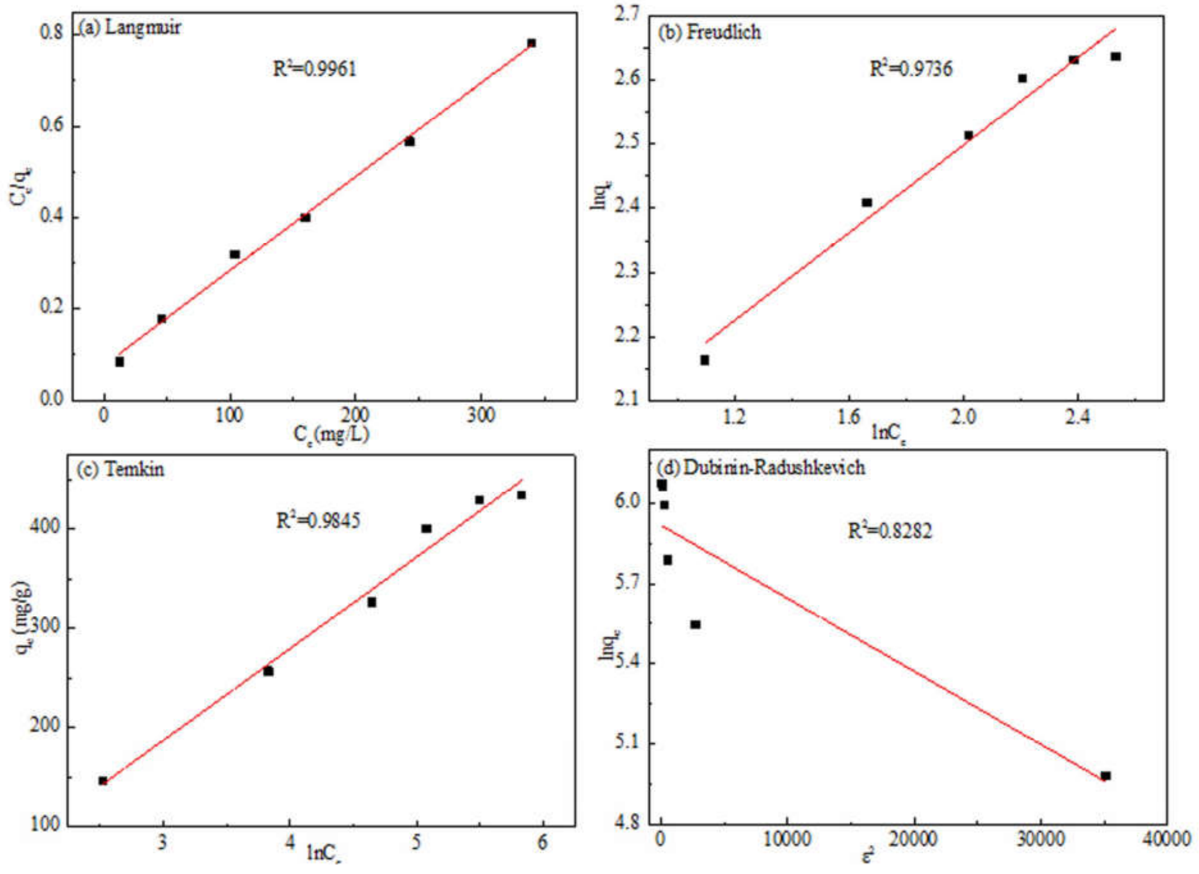

Figure S6. (a) Langmuir; **(b)** Freundlich; **(c)** Temkin; **(d)** Dubinin-Radushkevich isotherms for the Cu(II) adsorption on the surface of the nZVI/rGO magnetic nanocomposites.

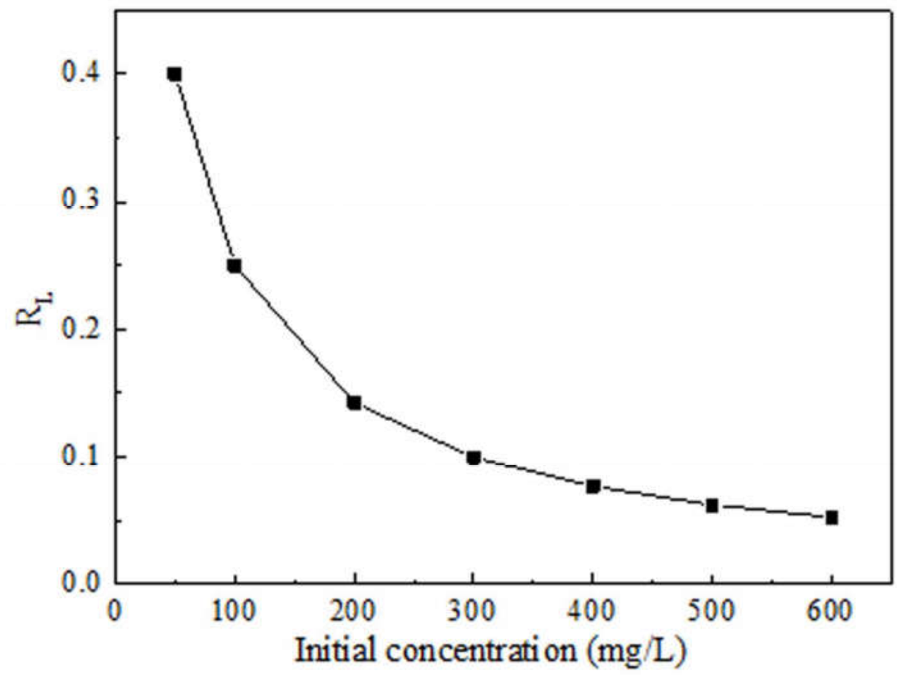

Figure S7. Relationship between initial Cu(II) concentration and R_L.

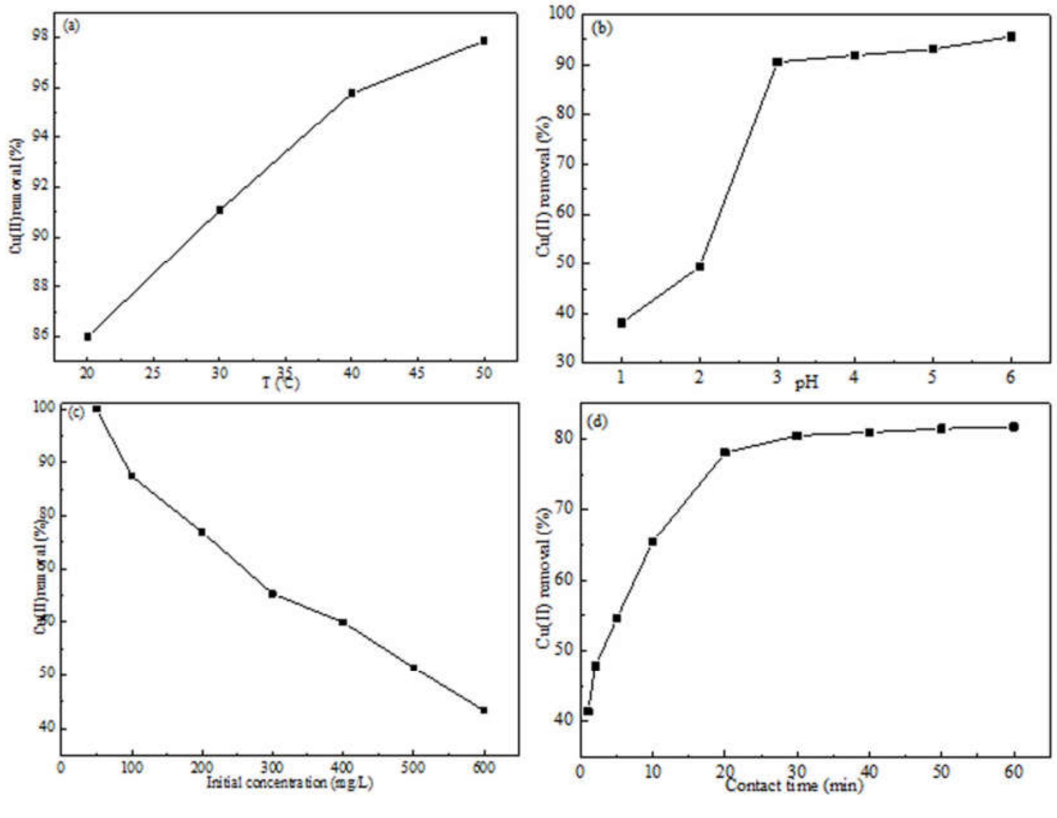

Figure S8. Effect of temperature on Cu(II) removal by nZVI/rGO composites: initial pH = 6.0; nZVI/rGO composites dosage = 30 mg; Cu(II) concentration = 100 mg/L; and time = 1h **(a)**. Effect of initial pH on Cu(II) removal by nZVI/rGO composites: Temperature = 20 °C; nZVI/rGO composites dose = 30 mg; Cu(II) concentration = 100 mg/L;

and time = 1h **(b).** Effect of initial Cu(II) concentration removal by nZVI/rGO composites: Temperature = 20 °C; initial pH =6.0; nZVI/rGO composites dose = 30 mg; and time = 1h **(c)**. Effect of contact time on Cu(II) removal by nZVI/rGO composites: Temperature = 20 °C; initial pH = 6.0; nZVI/rGO composites dose = 30 mg; and Cu(II) concentration = 100 mg/L **(d)**.

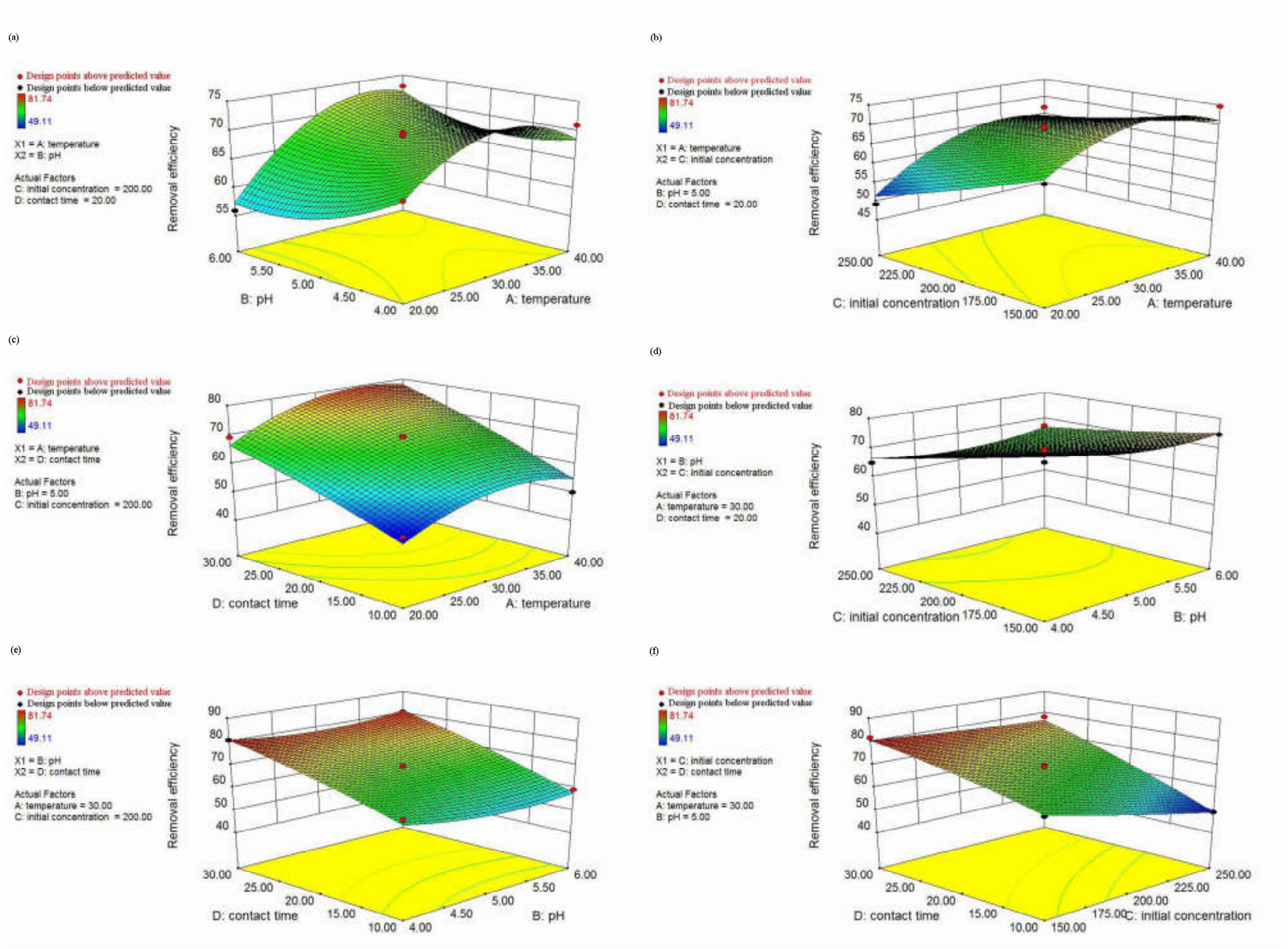

Figure S9. The 3D plots showing effect of temperature and initial pH **(a)**; temperature and initial Cu(II) concentration **(b)**; temperature and contact time **(c)**; pH and initial Cu(II) concentration **(d)**; pH and contact time **(e)**; and initial Cu(II) concentration and contact time on the Cu(II) removal **(f)**.

References

1. Fan, M.Y. *et al.* Synthesis and characterization of reduced graphene oxide-supported nanoscale zero-valent iron (nZVI/rGO) composites used for Pb(II) removal. *Materials* **9,** 687, https://doi.org/10.3390/ma9080687 (2016).

2. Jabeen, H., Kemp, K.C. & Chandra, V. Synthesis of nano zerovalent iron nanoparticles--graphene composite for the treatment of lead contaminated water. *J. Environ. Manage.* **130C,** 429-435, https://doi.org/10.1016/ j.jenvman. 2013.08.022 (2013).

3. Ali, R.M., Hamad, H.A., Hussein, M.M. & Malash, G.F. Potential of using green adsorbent of heavy metal removal from aqueous solutions: Adsorption kinetics, isotherm, thermodynamic, mechanism and economic analysis. *Ecol. Eng.* **91,** 317-332, https://doi.org/10.1016/j.ecoleng.2016.03.015 (2016).

4. Heibati, B. *et al.* Kinetics and thermodynamics of enhanced adsorption of the dye AR 18 using activated carbons prepared from walnut and poplar woods. *J. Mol. Liq.* **208,** 99-105, https://doi.org/10.1016/j.molliq.2015.03.057 (2015).

5. Alkan, M., Demirbas, O. & Dogan, M. Adsorption kinetics and thermodynamics of an anionic dye onto sepiolite. *Micropor. Mesopor. Mat.* **101,** 388-396, https://doi.org/10.1016/j.micromeso.2006.12.007 (2007).

6. Rong, X. *et al.* A facile hydrothermal synthesis, adsorption kinetics and isotherms to Congo Red azo-dye from aqueous solution of NiO/graphene nanosheets adsorbent. *J. Ind. Eng. Chem.* **26,** 354-363, ttps://doi.org/10.1016/ hj.jiec.2014.12.009 (2015).

7. Han, K., Kang, S.O., Park, S. & Park, H.S. Adsorption isotherms and kinetics of cationic and anionic dyes on three-dimensional reduced graphene oxide macrostructure. *J. Ind. Eng. Chem.* **21,** 1191-1196, https://doi.org/ 10.1016/j.jiec.2014.05.033 (2015).

8. Ngah, W.S.W. & Fatinathan, S. Adsorption of Cu(II) ions in aqueous solution using chitosan beads, chitosan-GLA beads and chitosan–alginate beads. *Chem. Eng. J.* **143,** 62-72, https://doi.org/10.1016/j.cej.2007.12.006 (2008).
